# Supplementary material for: Implications for Precision Accelerated Clinically Embedded Research (PACER): A novel technology-enabled approach to conducting minimal-risk research in outpatient community healthcare settings
Source: PLoS One. 2025 Apr 1;20(4):e0318533. doi: 10.1371/journal.pone.0318533 (PMC11961131; doi:10.1371/journal.pone.0318533)
Supplement: S1 File — (PDF) [file pone.0318533.s001.pdf]

Interview Guide

The following questions were asked of participants during the interviews. This version of the interview guide was created for clinicians. Analogous questions were asked of patients, research experts, and bioethics, with modifications made accordingly. For example, if clinician participants in this study were asked how they might feel about asking their (real-life) patient a question, patient participants in this study would be asked how they would feel if their (real-life) clinician asked them a question. Additionally, the interviewer could change the order of the questions, rephrase questions, and include unscripted prompts to request clarity or more explanation about a response as needed based on the flow of the interview. Questions that would not apply to patients (e.g., questions related to role and time in a particular role) were not included in interviews with patients.

Introductory questions

We will start with some questions about your previous experience with clinical research.

1. What is your experience with clinical research after residency?
2. What was your role?

Scenario 1 (A research question asked during the patient visit)

The first scenario is about a commonly prescribed medication (non-disease specific, for the purposes of this interview). A recent laboratory study suggested that this medication might increase the risk of knee joint problems. Researchers want to know if the incidence of knee disease is increased by this medication.

Now, let us suppose that on your schedule of patients for the day, there are several patients that the computer identifies as either being on this medication, or as control patients who are similar to patients who take the medication. When you see one of these patients, a pop-up alert in the EHR would prompt you to ask the patient if they have noticed pain or discomfort in their knee.

Assuming that you agreed to or are otherwise approved to get these types of pop-ups for research, the following questions apply/we'd like to ask the following…

Questions:

- How would you feel about asking your patient a specific question about knee pain, even though they came in for an entirely different reason?
- How would the addition of research questions to a patient visit impact patient care and your relationship with the patient?
- How do you feel about using time, even just a few minutes, during patients' clinic visits for this type of research?
- How would you feel about clinicians, clinic staff, or the clinic itself being compensated (financial or otherwise – e.g., training, resources, etc.) for taking part in this type of research?

- Are there non-financial forms of compensation, such as authorship, study partnership, public service, etc. that you would consider compelling?
- How would you feel about compensating patients for their participation in this type of research project (answering a research question during their visit)?
- How would adding a research question to the patient visit impact clinic workflow?
- What are your thoughts about the potential need for ongoing patient care depending on findings of the question/brief examination? For example, if the patient answers yes to knee pain, how would that affect patient care?
- What are your views about the EHR identifying patients to ask about their knee? Do you feel this affects patient privacy?
- How would the specific focus of a study (disease area, etc.) affect your willingness to participate in this kind of research?
- Which member of your care team do you think is most appropriate to ask these research questions?

Scenario 2 (Research question + physical examination)

Other questions about these scenarios:

- **Consent for research:** Participant consent is usually needed for research participation. Spending the whole clinic visit reviewing a consent form is not reasonable, but we know consent is necessary. There are many different forms/methods of participant consent to research that may be used, depending on the type of research. [Pass out the "Consent Options" on paper and conduct activity.]

- Do you think any of these options would work (and which one) for the research scenarios we discussed? Why did you choose that approach over the others? Are there any other ways (including those below or any not listed below) you might suggest consent be handled in these cases?

METHODS OF RESEARCH CONSENT TO PARTICIPATE

1. **Waiver of Consent:** The institution determined that no formal consent from the patient is needed. Just ask questions as part of your visit.
2. **Oral consent alone** - Provide a very brief explanation during the appointment and ask for the patient's oral consent, and then just ask the questions.
3. **Oral consent plus written information** - Provide brief explanation, ask for oral consent, then ask the questions. The patient receives written information about the study as part of their after-visit paperwork.
4. **Signed consent form** - Provide a consent form that explains the study, either before or during the clinic visit and ask the patient to review and sign it (electronically or on paper).
5. **Other approach to consent** – Please describe.

- **Data hold:** For any of the above, the study could hold the data for some amount of time (for instance 72 hours) to give the patient time to reconsider and "undo" the consent. If they ask and

undo, the data is deleted as if they never consented in the first place. Do you think the 72 hour "undo" is a good idea, and would it change your opinion about the best consent approach?

- **Uniform Consent:** Many medical centers have a uniform consent that says that patients may be contacted for research unless they specifically sign up for a "do not call" list. Do you think that this type of research should be included in the uniform consent to receive care, removing the need for separate consent for research questions/examinations like this during the patient visit?
- If a medical center did that, how patients should be informed of that fact? (e.g., by receiving information or by placing flyers in visible places in the medical office, or something else)?

General questions:

- What do you see as the major positive or negative aspects of this approach to research?
- Should there be a limit to the number or frequency of these kinds of questions? Is more than one question per clinical encounter acceptable? Should there be a limit to how often these occur for a given patient? (e.g., every visit or 1 of 5 visits)
- What about the number of visits per day for a given clinician? (i.e., would it be reasonable for a given clinician to have this kind of pop-up for all their patients or just a percentage of patients on a given day?)

Optional / Extra Time Questions:

- Collaborative Institutional Training Initiative (CITI) training is required for the conduct of research. Do you have a current CITI certification? If not, how would you feel about obtaining such a certification to be able to conduct this type of research?
- Suppose patients express interest in finding out the results of studies in which they participate. What are your thoughts about communicating study results to patients, particularly in cases where they might benefit from knowledge about the study result?
- How important do you feel it is for study results to be shared with clinicians and staff? How should results be shared?

Wrap-up:

- What question should I have asked that I did not ask?
- Are there any other barriers or concerns we have not discussed with integration of research into clinical practice?
- We are trying to anticipate the full set of benefits and downsides of this research technique. Is there anything very off-putting or problematic about the concept? Is there anything very exciting or beneficial about it?
- We'd like to talk to as many clinicians in as many multi-disciplinary roles as possible. Could you please suggest colleagues that we might interview? (Or you can share our info with them if you prefer not to give us their name)

Basic Demographic Information:

- What is your age?
- How do you describe your gender?
- How do you describe your race?

- How do you describe your ethnicity?
- What is your role (physician, nurse, staff member)?
- How many years have you been in this role?
- Address (for records)?
