# Supplementary figures and images for: Implications for Precision Accelerated Clinically Embedded Research (PACER): A novel technology-enabled approach to conducting minimal-risk research in outpatient community healthcare settings
Source: PLoS One. 2025 Apr 1;20(4):e0318533. doi: 10.1371/journal.pone.0318533 (PMC11961131; doi:10.1371/journal.pone.0318533)

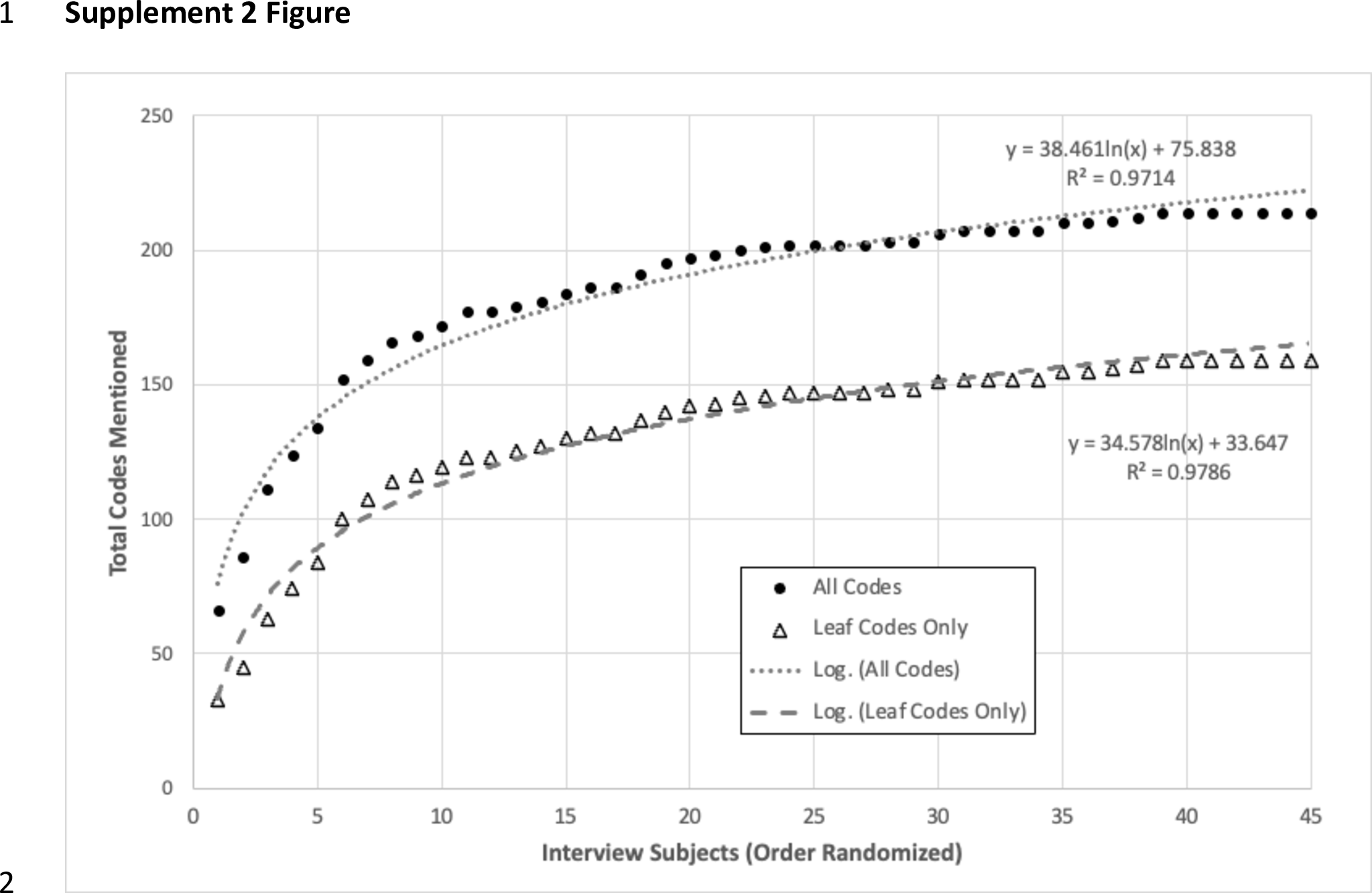

Supplement: S1 Fig — “All Codes” refers to topic codes in addition to the subcodes related to each topic. “Leaf Codes” include only the subcodes related to each topic. (TIF) [file pone.0318533.s002.tif]

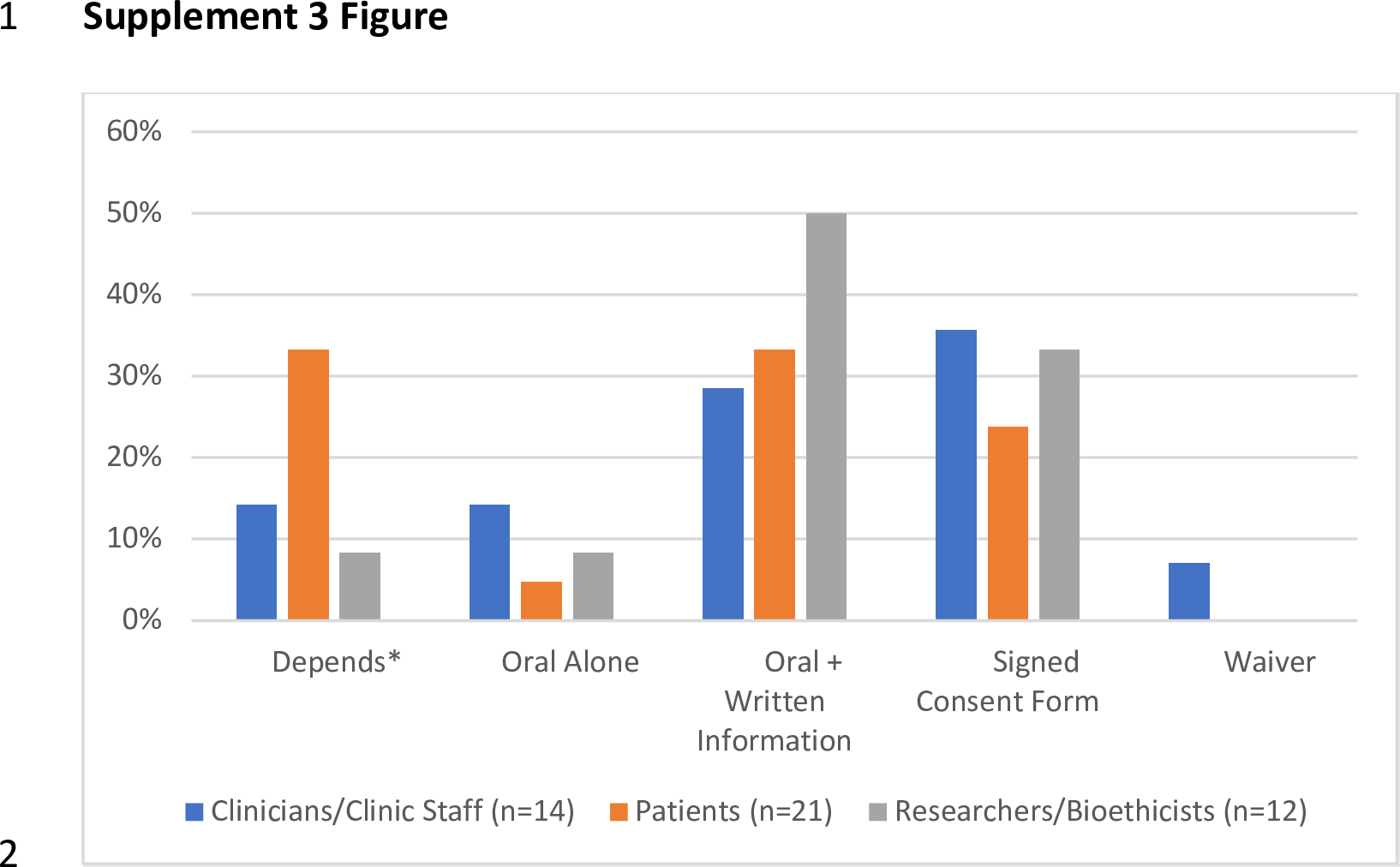

Supplement: S2 Fig — Asterisk indicates responses include those coded as “depends” and anyone who endorsed more than one response. One person did not endorse any of the consent types presented. (TIF) [file pone.0318533.s003.tif]

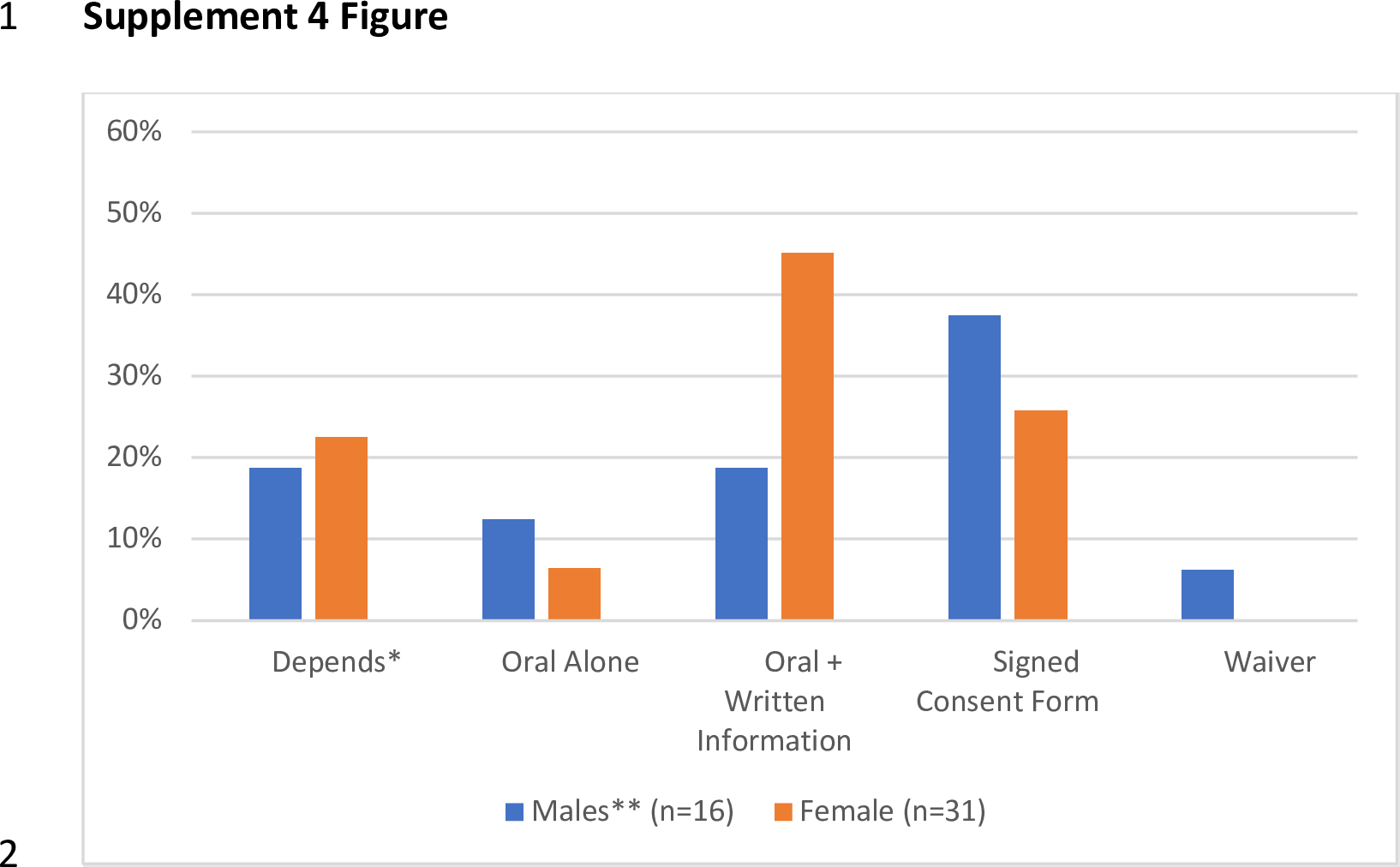

Supplement: S3 Fig — Single asterisk indicates responses include those coded as “depends” and anyone who endorsed more than one response. Double asterisk indicates data includes one person who self-identified as non-binary. One person did not endorse any of the consent types presented. (TIF) [file pone.0318533.s004.tif]
