# Supplementary material for: Implications for Precision Accelerated Clinically Embedded Research (PACER): A novel technology-enabled approach to conducting minimal-risk research in outpatient community healthcare settings
Source: PLoS One. 2025 Apr 1;20(4):e0318533. doi: 10.1371/journal.pone.0318533 (PMC11961131; doi:10.1371/journal.pone.0318533)
Supplement: Attachment — Submitted filename: PACER PLOS ONE Revisions Document final (1).docx [file pone.0318533.s006.docx]

**Part I: Reviewer Comments and Responses**

Each reviewer comment is numbered and the comments themselves are bolded. Our responses are below each comment and anything from text or additions to the text is italicized. All page and line numbers correspond to the new, edited version of the manuscript.

**Reviewer #1:**

**(1) The introduction needs to be revised as it is unclear. The discussion on PACER is not adequately explained—what it is and what it entails are not clear without referring to bibliographic entry "7," which makes the paper difficult to read. Consequently, the authors present isolated statements about the system, which make it difficult for readers to understand what this system entails.**

We thank you for your comment requesting additional details about the functioning and technical aspects of PACER, citing bibliographic entry 7. To that end, we have made the following additions to the introduction section of the manuscript. We explain in additional detail related to what we hope PACER can achieve and how we envision the PACER architecture would function. Please see the manuscript pages 3-4 lines 47-74 and below and we have added the appropriate reference for this citation:

*PACER’s mission is to maximize opportunities for broad and diverse participation in clinical research by expanding access to research studies and thereby improving the generalizability of results. This goal is shared by the National Institutes of Health, among other research organizations. The NIH’s newly appointed director has stated, “If we can build and support a research infra- structure that seamlessly integrates innovative science with routine clinical care, this will not only help us deliver high-quality results for people in a less resource-intensive way, but will allow us to get into diverse communities where we otherwise haven’t been.”[23] This aim is one our research team shares, and PACER is an embodiment of this aspiration. And with the increasing role of EHRs in clinical research, this new research infrastructure must interface with EHRs in non-AMC-affiliated and under-resourced clinics.*

*PACER leverages recent advances in technology called CDS hooks [11] to enable researchers to create and deliver to clinicians very short (under two minutes) research prompts—such as asking a question or doing a brief exam—into the clinical encounter. In doing so, PACER seeks to enable high-speed, real-time, scalable, minimal-risk research through rapid bi-directional transmission of information between a central hub and many distributed electronic health records (EHRs). This distributed approach allows research prompts to be sent to any participating clinician across the country, regardless of their location or which EHR they use. The responses to the prompts (e.g., answers to questions or results of the medical exam) would be documented in the medical record, thereby providing data about a symptom not typically asked about or physical finding not regularly included in a clinic visit. This additional information could also be returned to the central hub to rapidly answer research questions. The following example about how PACER might have been useful during the recent COVID-19 pandemic further explains PACER and its potential impact. After clinicians noted that some patients with COVID-19 experienced anosmia, PACER could have sent prompts to clinicians around the country whose patients had symptoms suggestive of COVID-19 requesting that the clinician inquire about anosmia. This information could have been paired with COVID-19 test results to rapidly confirm or deny an association between anosmia and COVID-19.*

**(2) Moreover, although the authors extol the system, these statements would benefit from better justification or even removal.**

Thank you for this important reminder to temper our statements. Throughout the paper, we are careful to use terms such as “could,” “hope,” “seek,” or “maybe.” We have edited the manuscript to moderate our statements and provide a more balanced perspective, and we note PACER’s limitations.

Below, we list a few examples of how we moderated our statements.

1. On page 4 line 87, we deleted “*enormous*” regarding PACER’s potential.

2. On page 22 line 589, we changed “even” to “might” in the context of PACER’s potential to support improved resource allocation.

3. On page 32 line 854, we have removed the modifier, “enthusiastically” to describe our participants’ responses to PACER’s potential benefits.

**(3) Were the participants adequately prepared before administering the questionnaire? Were they informed, even in broad terms, about the issues at hand? Was any information provided on this topic?**

Thank you for these questions—participant preparation is an important issue that we appreciate clarifying. Participants were informed in broad terms of the subject of the study and the most salient features of the discussion through an information sheet provided to potential participants prior to interviews. Additionally, at the commencement of the interview, the interviewer gave an overview of PACER to contextualize the discussion. We added clarifying text on pages 7-8 lines 287-297 and here it is below:

*Prior to confirming their involvement in the study, potential interviewees received an information sheet, sent electronically via email. The information sheet included a brief description PACER, of the study’s aims, the risks and benefits of participation, and* *study and institutional contact information. When the interviews commenced, the interviewer gave a brief description of PACER and how PACER would function and offered the participants the opportunity to ask their own questions prior to the interview questions.*

**(4)** **Please provide a better justification for the following statement: "However, obtaining written informed consent from participating patients could prolong the clinic encounter—reducing efficiency and potentially affecting participation" (line 483).**

We apologize for the lack of clarity. The issue of consent, and how the consent process would interact with clinical workflow, is important. As is typical in surveys of research subject attitudes about consent, there was a heterogeneity of opinion. In the results we noted,

“*Some comments reflected the concern that truncating the consent process (e.g., requiring only oral permission) risks harming the process’s integrity and inhibiting patients from making well-informed choices. However other participants described how obtaining informed consent has its own associated burden*” (page 29 line 761-765).

In addition, in Table 2, Domain 2 Consent, Subtheme 1b Clinic factors, we report the quote,

“*A signed consent form adds time, complexity, interruption ...if the written information could be passed off like in the MA check right, and just say, ‘Hey, we're doing a little—we're doing some research on a medication you're taking. Here's just some information, right if you're comfortable, the provider may ask some questions, and you don't have to say, if you want to, and here's a little bit of information you can read while you wait for the provider to come into the room’ that's pretty fluid*” (12).

To further clarify this topic, we have changed the sentence to the following on page 29 lines 764-765.

*However, other participants noted that obtaining written informed consent could prolong the clinical encounter—reducing efficiency and potentially affecting participation.*

**(5) The results are not well aligned with the description. Given the description of the participants, were differences in viewpoints considered?**

In retrospect, we realize that the opening of the discussion section may not have captured the overall sense of the results. We have reworked this section including adding several clarifying statements including on page 28 at line 727-735:

*Most reactions to PACER were positive. Participants indicated that PACER could improve clinical research processes and increase research participation, as well as advancing scientific knowledge.* *At the same time, the results demonstrated diverse views about implementing various aspects of PACER methodology. We discovered a diversity of opinions within each group of respondents. Although the responses were not amenable to a formal statistical analysis, we did not identify obvious trends within or among each category of interviewee. Thus, there was no unanimity regarding a single optimal design for a PACER implementation, and future implementers will need to weigh the various opinions.*

We also added at page 35 lines 908-911:

*Additionally, we acknowledge that we may have failed to capture the full gamut of possible viewpoints as some participants expressed differing or contradictory opinions within one interview and certain demographic groups were insufficiently represented in this preliminary study.*

**(6)** **Overall, I find it challenging to understand the utility of the work, and the target audience of the article is not clear.**

Thank you for bringing up the important issues of the utility of this work and our target audience. The primary goals of PACER are to increase the pace of and expand the access to biomedical research. In this particular work, we are exploring our interviewees’ viewpoints on a number of topics related to PACER’s design and implementation. To better describe the utility of the work, we have added the following to the introduction on page 5 at lines 217-219:

*By exploring and describing diverse viewpoints related to PACER, and by identifying potential challenges imposed by PACER a priori, we intend for this work to inform the creation of guidelines for future PACER implementation.*

We feel these data will inform researchers interested in implementing PACER and clinicians and patients who may be involved in research that uses PACER methodology. To further clarify this we have included the following to the end of the first paragraph in the discussion on page 28-29 at lines 744-756.

*We believe that this study of participants’ perceptions of PACER and its ethical implications, as well as how to implement PACER, can help guide the wider research and bioethics communities—including researchers, bioethicists, institutional review boards (IRBs), and others—who may wish to implement PACER*.

**Reviewer #2:**

**(1) Thank you for the opportunity to review this paper that qualitatively seeks to understand the ramifications of a distributive methodology to conduct low risk (human subject risk) research. This process may help in increasing equitable burden of research while still holding to the justice principles. Overall this was a well done description of interview methodology and provides a clear path to data collection and distillation.**

Thank you for these positive comments; we appreciate your vote of confidence and your sharing our vision.

**(2) The only methodological concern I had was I was not clear in how the interviews were conducted, e.g. were they individual or group settings. if in groups, were the groups mixed with respect to the types of participants (e.g. ethicists, providers, regulatory)**

Thank you so much for bringing this clarification question to our attention. All interviews were conducted individually (i.e., one-on-one) via teleconferencing (over Zoom). To make this clearer, we have added the following phrase--”one-on-one"--to the manuscript—please see page 6 line 191 and also below:

*We conducted one-on-one, qualitative semi-structured interviews with patients, clinicians, research experts, and bioethicists between February and August 2022*.

**(3) I also have a generalizability concern, subjects were restricted to the Chicago area. This concern is addressed in the limitations discussion, however, authors admit that the participants mirrored the Chicago mixture of society, but that does not necessarily mirror the greater American population. I would ask that authors make this limitation a little stronger instead of minimizing this area.**

Generalizability is one of our top priorities, so we thank you for your comment on this subject. We more strongly acknowledge this limitation below, along with explaining our reasoning (that we hope that Chicago is a good starting off point on which to base future endeavors and that saturation from our Zipf analysis was reached). Please see page 36 lines 875-879 and below:

*Given that Chicagoland is a highly diverse region, we felt it a good place to start this exploration of people’s view about PACER. Even so, it is clear that future studies with larger geographic coverage, and inclusion of ethnic groups not well represented in Chicagoland, e.g. Native American, Alaska Native, Native Hawaiian, and Pacific Islander, are needed.*

**(4) Overall this is a well done and well written report. Thank you for your contribution.**

Thank you. We so appreciate the opportunity to resubmit this manuscript and feel it is much improved as a result of the requested revisions.

**Part II: Editor Comments and Responses**

The journal’s editor comments are listed below, and each comment is followed by a response. They are numbered for convenience, and the first sentence of each comment is bolded.

Journal Requirements:

**1.** **When submitting your revision, we need you to address these additional requirements.**

**Please ensure that your manuscript meets PLOS ONE's style requirements, including those for file naming. The PLOS ONE style templates can be found at** 
[https://journals.plos.org/plosone/s/file?id=wjVg/PLOSOne_formatting_sample_main_body.pdf](https://urldefense.com/v3/__https:/journals.plos.org/plosone/s/file?id=wjVg*PLOSOne_formatting_sample_main_body.pdf__;Lw!!M0gZt9BicGig!juHS1kIInOdK9-rGUGEY9ueW-G2IhjQ3-sBpbX8n0Q3AODOOxvPyvUELoGE4jScpLMej-fq6GuJ2GdN7YCJ6-5g$) and 
[https://journals.plos.org/plosone/s/file?id=ba62/PLOSOne_formatting_sample_title_authors_affiliations.pdf](https://urldefense.com/v3/__https:/journals.plos.org/plosone/s/file?id=ba62*PLOSOne_formatting_sample_title_authors_affiliations.pdf__;Lw!!M0gZt9BicGig!juHS1kIInOdK9-rGUGEY9ueW-G2IhjQ3-sBpbX8n0Q3AODOOxvPyvUELoGE4jScpLMej-fq6GuJ2GdN7o4Oflow$)

Thank you so much for sharing these helpful resources with us. We are very grateful to have this information to consult. To that end, we have done the following:

1. We have deleted the address listed for the corresponding author and just kept the email address as instructed.

2. Headings: In keeping with sentence capitalization, we changed the “m” in “methods” in the heading in line 121 from uppercase to lowercase as the guide requires. On the same page at line 131, we changed the “g” in “Interview guide” from uppercase to lowercase. On the following page (at line 164), we changed from uppercase to lowercase several letters in the heading, “Participant recruitment and data collection.” We changed the “a” in the heading, “Data analysis” at line 194 from uppercase to lowercase. At line 261, we changed the heading “Impact on clinical research” to lowercase from uppercase. We also changed the “t” in type from uppercase to lowercase in heading “Consent type” at line 285. At line 321, we changed the “c” in “Consent” from uppercase to lowercase. At line 335, we changed the uppercase “P” to lowercase. At line 377, there is a heading with a slash in it. We changed the “c” in compensation from uppercase to lowercase, but we’re unsure if we are be permitted to keep the slash or the capitalization next to it. We also changed the “c” in compensation from uppercase to lowercase at lines 377 and 395. At line 409, we changed from uppercase to lowercase the “p” and “o” in “Impacts on people and organizations.” The “c” in “considerations” was changed from uppercase to lowercase at line 480. We also changed to lowercase letters in the heading on line 501, “Post-study contact.” The “q” in question at line 535 is also now lowercase. At line 549, the “t” in “topics” was changed to lowercase from uppercase.

We also changed the headings in our tables to match this style.

**2.** **Thank you for stating the following in the Acknowledgments Section of your manuscript**: 
Research reported in this publication was supported, by The Greenwall Foundation and the National Institutes of Health's National Center for Advancing Translational Sciences, Grant Number UL1TR001422. The content is solely the responsibility of the authors and does not necessarily represent the official views of the Greenwall Foundation or the National Institutes of Health.

We note that you have provided funding information that is not currently declared in your Funding Statement. However, funding information should not appear in the Acknowledgments section or other areas of your manuscript. We will only publish funding information present in the Funding Statement section of the online submission form. 
Please remove any funding-related text from the manuscript and let us know how you would like to update your Funding Statement. Currently, your Funding Statement reads as follows: 
Author who received award: Justin Starren
Funder: The Greenwall Foundation
URL: [https://greenwall.org/](https://urldefense.com/v3/__https:/greenwall.org/__;!!M0gZt9BicGig!juHS1kIInOdK9-rGUGEY9ueW-G2IhjQ3-sBpbX8n0Q3AODOOxvPyvUELoGE4jScpLMej-fq6GuJ2GdN7BSr4gu0$)
The funder did not play any role in the study design, data collection and analysis, decision to publish, or preparation of the manuscript.

Author who received funding: Justin Starren
Grant Number: UL1TR001422
Name of Funder: National Institutes of Health's National Center for Advancing Translational Sciences
The funder did not play any role in the study design, data collection and analysis, decision to publish, or preparation of the manuscript

Please include your amended statements within your cover letter; we will change the online submission form on your behalf.

Thank you very much for bringing the acknowledgements section to our attention. We understand that financial information is not supposed to be included in the Acknowledgements section, so we have removed all of the funding information from it. In the Funding Statement section, we have reformatted the financial information to fit the journal’s requirements.

**3.** **Thank you for stating the following in the Competing Interests section**: 
I have read the journal's policy and the authors of this manuscript have the following competing interests:
Dr. Kelly Michelson has received funding for other work from the Patient Centered Outcomes Research Institute and the National Institutes of Health
Mohammad Hosseini was supported by the National Institutes of Health’s National Center for Advancing Translational Sciences (UL1TR001422)
Matthew Baumann's employment is supported by a grant from the National Evaluation Center of the Network of the National Library of Medicine (NEC/NNLM)

We note that one or more of the authors are employed by a commercial company.

a. Please provide an amended Funding Statement declaring this commercial affiliation, as well as a statement regarding the Role of Funders in your study. If the funding organization did not play a role in the study design, data collection and analysis, decision to publish, or preparation of the manuscript and only provided financial support in the form of authors' salaries and/or research materials, please review your statements relating to the author contributions, and ensure you have specifically and accurately indicated the role(s) that these authors had in your study. You can update author roles in the Author Contributions section of the online submission form.

Please also include the following statement within your amended Funding Statement. 
“The funder provided support in the form of salaries for authors, but did not have any additional role in the study design, data collection and analysis, decision to publish, or preparation of the manuscript. The specific roles of these authors are articulated in the ‘author contributions’ section.”
If your commercial affiliation did play a role in your study, please state and explain this role within your updated Funding Statement.

b. Please also provide an updated Competing Interests Statement declaring this commercial affiliation along with any other relevant declarations relating to employment, consultancy, patents, products in development, or marketed products, etc.

Within your Competing Interests Statement, please confirm that this commercial affiliation does not alter your adherence to all PLOS ONE policies on sharing data and materials by including the following statement: "This does not alter our adherence to  PLOS ONE policies on sharing data and materials.” (as detailed online in our guide for authors [http://journals.plos.org/plosone/s/competing-interests](https://urldefense.com/v3/__http:/journals.plos.org/plosone/s/competing-interests__;!!M0gZt9BicGig!juHS1kIInOdK9-rGUGEY9ueW-G2IhjQ3-sBpbX8n0Q3AODOOxvPyvUELoGE4jScpLMej-fq6GuJ2GdN7wkPjYtk$)) . If this adherence statement is not accurate and  there are restrictions on sharing of data and/or materials, please state these. Please note that we cannot proceed with consideration of your article until this information has been declared.

Please include both an updated Funding Statement and Competing Interests Statement in your cover letter. We will change the online submission form on your behalf.

We have carefully reviewed all of our funding information and the affiliation for each author. We did not find any commercial affiliations. We feel confident about the accuracy of our investigation. Our examination of funders reveals the following sources:

- The Greenwall Foundation (a nonprofit foundation that funds bioethics research)
- National Institutes of Health's National Center for Advancing Translational Sciences (Grant Number: UL1TR001422)
- National Evaluation Center of the Network of the National Library of Medicine
- Patient Centered Outcomes Research Institute
- The National Institutes of Health

None of these funding sources is commercial; all are federal, with the exception of the Greenwall Foundation. These funders did not have any role in the study design, data collection and analysis, decision to publish, or preparation of the manuscript.

We wrote the journal to inquire about how to proceed. The response is pasted below:

“From time to time, funding sources associated with a manuscript are incorrectly identified as commercial in nature, necessitating the provision of an updated Financial Disclosure statement which includes the required compliance statement. In circumstances such as this, where the funding sources are not commercial and the correlated change is not required, we ask that authors just articulate this in their response to the revision decision they received. To do so, please include the information you have provided here [it is above] in the Journal Office requirements section of your Response to Reviewers document, submitted alongside your revised manuscript. Your response will be evaluated by our vendors and staff, who will determine if any additional follow up is needed.”

Here are our amended Funding Statement, a Role of Funders Statement, an amended Competing Interests Statement, and we have reviewed the online Author Contributions form.

Financial Disclosure Statement (Revised):

1. J.S. was awarded a grant from The Greenwall Foundation

This grant funded the work of the following members of the study team on this project: J.S., K.M., E.F., S.S., T.W., and A.V.

The funders had no role in study design, data collection and analysis, decision to publish, or preparation of the manuscript

Grant number: N//A (The is no individual number associated with this grant)

URL: [https://greenwall.org/](https://urldefense.com/v3/__https:/greenwall.org/__;!!M0gZt9BicGig!juHS1kIInOdK9-rGUGEY9ueW-G2IhjQ3-sBpbX8n0Q3AODOOxvPyvUELoGE4jScpLMej-fq6GuJ2GdN7BSr4gu0$)

2. J.S., has received a grant to support their work from National Institutes of Health's National Center for Advancing Translational Sciences—all of these individuals are included on this same grant

This grant has funded the work of K.M., M.H., M.B.

The funders had no role in study design, data collection and analysis, decision to publish, or preparation of the manuscript. This work does not represent the views of the NIH, NCATS or US government.

Grant number: UL1TR001422

URL: https://ncats.nih.gov/funding/overview

Competing Interest Statement (Revised):

Aside from one listed below, the authors attest that that no other grants, funding sources, or affiliations are related to, interfere with, or could reasonably be perceived as interfering with, the full and objective presentation, peer review, editorial decision-making, or publication of this particular research study. Several authors have received funding from a variety of sources for different research projects. We wish to be in full compliance with journal requirements. Should you wish to access information about these other interests, our team is happy to share these details; they are not listed in this disclosure because they are not directly related to the presentation, review, or publication of this manuscript. Aside from one listed below, the authors attest that that no other grants, funding sources, or affiliations are related to, interfere with, or could reasonably be perceived as interfering with, the full and objective presentation, peer review, editorial decision-making, or publication of this particular research study. Several authors have received funding from a variety of sources for different research projects. We wish to be in full compliance with journal requirements. Should you wish to access information about these other interests, our team is happy to share these details; they are not listed in this disclosure because they are not directly related to the presentation, review, or publication of this manuscript. We have verified that we do not have any similar projects, nor do we have any projects that have a direct bearing on this study or its publication. Therefore, after careful evaluation, we have not found interests that appear to “reasonably interfere.”

These items are other funding sources for research team members:

1. M.B. has received salary support funding from National Evaluation Center (NEC) for the Network of the National Library of Medicine (NNLM)

The funders had no role in study design, data collection and analysis, decision to publish, or preparation of the manuscript

Grant number: 5U24LM013751-04

URL: <https://www.nnlm.gov/funding>

2. M.H. is also supported by a grant from National Institutes of Health's National Center for Advancing Translational Sciences

The funders have not played a role in the design, analysis, decision to publish, or preparation of the manuscript. This work does not represent the views of the NIH, NCATS or US government.

Grant number: UM1TR005121

URL: https://ncats.nih.gov/funding/overview

M.B. has no additional competing interests as his stock and device ownership has no relationship to this project.

K.M. has no competing interests as none of her other financial awards have a relationship with this project.

J.S. has no competing interests as none of his other financial awards have a relationship with this project.

E.F. has no competing interests.

T.W. has no competing interests as none of her other financial awards have a relationship with this project.

M.H. has no competing interests as none of his other financial awards have a relationship with this project.

R.S. has no competing interests

A.V. has no competing interests

4. **In this instance it seems there may be acceptable restrictions in place that prevent the public sharing of your minimal data. However, in line with our goal of ensuring long-term data availability to all interested researchers, PLOS’ Data Policy states that authors cannot be the sole named individuals responsible for ensuring data access (**[**http://journals.plos.org/plosone/s/data-availability#loc-acceptable-data-sharing-methods**](https://urldefense.com/v3/__http:/journals.plos.org/plosone/s/data-availability*loc-acceptable-data-sharing-methods__;Iw!!M0gZt9BicGig!juHS1kIInOdK9-rGUGEY9ueW-G2IhjQ3-sBpbX8n0Q3AODOOxvPyvUELoGE4jScpLMej-fq6GuJ2GdN7RzssKcw$)**).**

**Data requests to a non-author institutional point of contact, such as a data access or ethics committee, helps guarantee long term stability and availability of data. Providing interested researchers with a durable point of contact ensures data will be accessible even if an author changes email addresses, institutions, or becomes unavailable to answer requests.**

**Before we proceed with your manuscript, please also provide non-author contact information (phone/email/hyperlink) for a data access committee, ethics committee, or other institutional body to which data requests may be sent. If no institutional body is available to respond to requests for your minimal data, please consider if there any institutional representatives who did not collaborate in the study, and are not listed as authors on the manuscript, who would be able to hold the data and respond to external requests for data access? If so, please provide their contact information (i.e., email address). Please also provide details on how you will ensure persistent or long-term data storage and availability.**

We are committed to making the deidentified transcripts publicly available through a publicly accessible repository under a Data Use Agreement. We have identified a repository with whom we would like to deposit our data, and we are actively working to implement this solution. In correspondence with you, we were informed that we have the option to select a checkbox that reads "Tick here if the URLs/accession numbers/DOIs will be available only after acceptance of the manuscript for publication so that we can ensure their inclusion before publication” during the submission process. Thus, our updated Data Availability Statement outlines that we will make the study’s limited data set will be available upon acceptance. Here is an excerpt of the helpful, thorough guidance that we received:

“PLOS ONE does provide a mechanism for authors who wish to proceed with the peer review process before they can make their data publicly available. In these instances, we ask that authors indicate that their manuscript's minimal data set will be provided when their manuscript is accepted for publication. In the submission system, adjacent to the field where you can enter your Data Availability statement, there is a checkbox that reads "Tick here if the URLs/accession numbers/DOIs will be available only after acceptance of the manuscript for publication so that we can ensure their inclusion before publication.". Please check this box when submitting your revised manuscript. You can also provide an updated Data Availability statement which indicates you are currently working to find an acceptable public repository for your data, but, in order to facilitate the peer review process, you will provide the repository URL and other information when your manuscript is accepted.”

**5. Please include your full ethics statement in the ‘Methods’ section of your manuscript file. In your statement, please include the full name of the IRB or ethics committee who approved or waived your study, as well as whether or not you obtained informed written or verbal consent. If consent was waived for your study, please include this information in your statement as well.**

We appreciate the opportunity to clarify this important item. We added the following sentence as an ethics statement to our methods. We hope that this is in compliance with the journal’s requirements. At page 6 on lines 127-129, it reads:

“*No funders had a role in the study design, data collection and analysis, decision to publish, or preparation of the manuscript*.”

Thank you for bringing up this important point. In our manuscript, we include information in the methods section about the name of the IRB who waived our study and that it was deemed exempt thus not requiring a consent form. We also mention that we sought verbal consent prior to recording interviews. This appears to meet with the requirements as it includes the details mentioned above. We are more than happy to add any additional information or change the formatting as necessary. Below is the text (in the manuscript on page 5 at lines 223-230) including all of these data.

*We conducted one-on-one, qualitative semi-structured interviews with patients, clinicians, research experts, and bioethicists between February 1 2022 and August 31 2022. Verbal consent to participate in this study and for the interviews to be audio recorded was obtained by the interviewer prior to starting the recording of interviews. This study was determined to be exempt by the Northwestern University and the Ann & Robert H. Lurie Children’s Hospital Institutional Review Boards (NU IRB* STU00215847 *and Lurie IRB 2021-4774).*
